# Bis(2-cyclo­hexyl­imino­methyl-4,6-disulfanylphenolato)zinc(II)

**DOI:** 10.1107/S1600536809032048

**Published:** 2009-08-19

**Authors:** Wu Chen, Di Xu, Lian Liu, Qing-Fu Zeng

**Affiliations:** a Engineering Research Center for Clean Production of Textile Dyeing and Printing, Ministry of Education, Wuhan 430073, People’s Republic of China

## Abstract

In the title complex, [Zn(C_13_H_16_NOS_2_)_2_], the Zn^II^ ion is four-coordinated by two *N*,*O*-bidentate Schiff base ligands, resulting in a distorted *trans*-ZnN_2_O_2_ square-planar geometry for the metal ion.

## Related literature

For background to Schiff bases as ligands, see: Shi *et al.* (2008 ▶); Xu *et al.* (2009 ▶). For reference structural data, see: Allen *et al.* (1987 ▶).
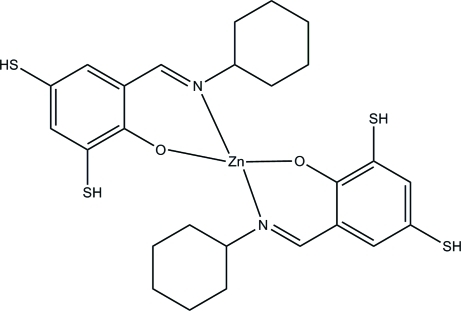


## Experimental

### 

#### Crystal data

[Zn(C_13_H_16_NOS_2_)_2_]
*M* *_r_* = 598.15
Monoclinic, 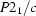

*a* = 15.031 (4) Å
*b* = 12.663 (3) Å
*c* = 14.182 (4) Å
β = 91.735 (15)°
*V* = 2698.1 (11) Å^3^
*Z* = 4
Mo *K*α radiation
μ = 1.25 mm^−1^
*T* = 296 K
0.30 × 0.20 × 0.20 mm

#### Data collection

Enraf–Nonius CAD-4 diffractometer
Absorption correction: ψ scan (North *et al.*, 1968 ▶) *T*_min_ = 0.706, *T*_max_ = 0.789
14026 measured reflections
4746 independent reflections
3839 reflections with *I* > 2σ(*I*)
*R*_int_ = 0.027
3 standard reflections every 200 reflections intensity decay: 1%

#### Refinement

*R*[*F* ^2^ > 2σ(*F* ^2^)] = 0.045
*wR*(*F* ^2^) = 0.141
*S* = 1.04
4746 reflections
320 parameters
6 restraints
H-atom parameters constrained
Δρ_max_ = 0.58 e Å^−3^
Δρ_min_ = −0.56 e Å^−3^

### 

Data collection: *CAD-4 Software* (Enraf–Nonius, 1989 ▶); cell refinement: *CAD-4 Software*; data reduction: *XCAD4* (Harms & Wocadlo, 1995 ▶); program(s) used to solve structure: *SHELXS97* (Sheldrick, 2008 ▶); program(s) used to refine structure: *SHELXL97* (Sheldrick, 2008 ▶); molecular graphics: *SHELXTL* (Sheldrick, 2008 ▶); software used to prepare material for publication: *SHELXTL*.

## Supplementary Material

Crystal structure: contains datablocks global, I. DOI: 10.1107/S1600536809032048/hb5044sup1.cif

Structure factors: contains datablocks I. DOI: 10.1107/S1600536809032048/hb5044Isup2.hkl

Additional supplementary materials:  crystallographic information; 3D view; checkCIF report

## Figures and Tables

**Table:** 

Zn1—N1	1.971 (3)
Zn1—N2	1.971 (3)
Zn1—O1	1.899 (2)
Zn1—O2	1.886 (2)

**Table:** 

O2—Zn1—O1	151.46 (13)
O2—Zn1—N1	94.46 (11)
O1—Zn1—N1	93.98 (11)
O2—Zn1—N2	90.96 (12)
O1—Zn1—N2	92.96 (12)
N1—Zn1—N2	154.63 (14)

**Fig. 1.**
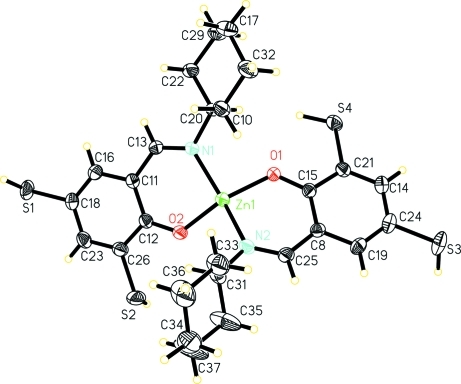
The molecular structure of (I) showing 30% probability displacement ellipsoids.

## supplementary crystallographic information

### Comment

There has been much research interest in Schiff base metal complexes due to their molecular architectures and biological activities (Shi *et al.*, 2008; Xu *et al.*, 2009). In this work, we report here the crystal structure of the title compound, (I). In (I), all bond lengths are within normal ranges (Allen *et al.*, 1987) (Fig. 1). The Zn(II) is four-coordinated in a distort square-planar configuration by two N atoms and two O atoms of the Schiff base ligand (Table 1).

### Experimental

A mixture of 2-hydroxy-3,5-disulfanylbenzaldehyde (372 mg, 2 mmol), cyclohexanamine (198 mg, 2 mmol) and ZnCl2 (1 mmol, 134 mg) in methanol (10 ml) was stirred for 1 h. After keeping the filtrate in air for 7 d, colourless blocks of (I) were formed.

### Refinement

All H atoms were positioned geometrically (C—H = 0.93–0.97 Å, S—H = 1.20Å) and refined as riding, with *U*_iso_(H) = 1.2*U*_eq_(carrier) or 1.5*U*_eq_(methyl C).

### Crystal data

**Table:**

[Zn(C_13_H_16_NOS_2_)_2_]	*F*(000) = 1248
*M**_r_* = 598.15	*D*_x_ = 1.473 Mg m^−^^3^
Monoclinic, *P*2_1_/*c*	Mo *K*α radiation, λ = 0.71073 Å
Hall symbol: -P 2ybc	Cell parameters from 25 reflections
*a* = 15.031 (4) Å	θ = 9–12°
*b* = 12.663 (3) Å	µ = 1.25 mm^−^^1^
*c* = 14.182 (4) Å	*T* = 296 K
β = 91.735 (15)°	Block, colourless
*V* = 2698.1 (11) Å^3^	0.30 × 0.20 × 0.20 mm
*Z* = 4	

### Data collection

**Table:**

Enraf–Nonius CAD-4 diffractometer	3839 reflections with *I* > 2σ(*I*)
Radiation source: fine-focus sealed tube	*R*_int_ = 0.027
graphite	θ_max_ = 25.0°, θ_min_ = 2.1°
ω/2θ scans	*h* = −17→11
Absorption correction: ψ scan (North *et al.*, 1968)	*k* = −15→14
*T*_min_ = 0.706, *T*_max_ = 0.789	*l* = −16→16
14026 measured reflections	3 standard reflections every 200 reflections
4746 independent reflections	intensity decay: 1%

### Refinement

**Table:**

Refinement on *F*^2^	Primary atom site location: structure-invariant direct methods
Least-squares matrix: full	Secondary atom site location: difference Fourier map
*R*[*F*^2^ > 2σ(*F*^2^)] = 0.045	Hydrogen site location: inferred from neighbouring sites
*wR*(*F*^2^) = 0.141	H-atom parameters constrained
*S* = 1.03	*w* = 1/[σ^2^(*F*_o_^2^) + (0.0766*P*)^2^ + 2.9389*P*] where *P* = (*F*_o_^2^ + 2*F*_c_^2^)/3
4746 reflections	(Δ/σ)_max_ = 0.013
320 parameters	Δρ_max_ = 0.58 e Å^−^^3^
6 restraints	Δρ_min_ = −0.56 e Å^−^^3^

### Special details

**Table:**

Geometry. All e.s.d.'s (except the e.s.d. in the dihedral angle between two l.s. planes) are estimated using the full covariance matrix. The cell e.s.d.'s are taken into account individually in the estimation of e.s.d.'s in distances, angles and torsion angles; correlations between e.s.d.'s in cell parameters are only used when they are defined by crystal symmetry. An approximate (isotropic) treatment of cell e.s.d.'s is used for estimating e.s.d.'s involving l.s. planes.
Refinement. Refinement of *F*^2^ against ALL reflections. The weighted *R*-factor *wR* and goodness of fit *S* are based on *F*^2^, conventional *R*-factors *R* are based on *F*, with *F* set to zero for negative *F*^2^. The threshold expression of *F*^2^ > σ(*F*^2^) is used only for calculating *R*-factors(gt) *etc*. and is not relevant to the choice of reflections for refinement. *R*-factors based on *F*^2^ are statistically about twice as large as those based on *F*, and *R*- factors based on ALL data will be even larger.

### Fractional atomic coordinates and isotropic or equivalent isotropic displacement parameters (Å^2^)

**Table:**

	*x*	*y*	*z*	*U*_iso_*/*U*_eq_	
C8	0.8408 (2)	0.3618 (3)	0.5588 (2)	0.0389 (8)	
C10	0.6332 (2)	0.2572 (3)	0.8300 (3)	0.0393 (8)	
H10	0.6150	0.3068	0.7800	0.047*	
C11	0.6548 (2)	−0.0326 (3)	0.7702 (2)	0.0389 (8)	
C12	0.7190 (2)	−0.0464 (3)	0.6999 (3)	0.0399 (8)	
C13	0.6325 (2)	0.0695 (3)	0.8077 (3)	0.0407 (8)	
H13	0.5907	0.0704	0.8548	0.049*	
C14	0.7450 (3)	0.5405 (3)	0.5026 (3)	0.0465 (9)	
H14	0.7134	0.6007	0.4845	0.056*	
C15	0.7538 (2)	0.3763 (3)	0.5942 (2)	0.0390 (8)	
C16	0.6116 (3)	−0.1202 (3)	0.8080 (3)	0.0457 (9)	
H16	0.5697	−0.1103	0.8542	0.055*	
C17	0.5281 (3)	0.3530 (4)	0.9314 (3)	0.0577 (11)	
H17A	0.5101	0.3975	0.8786	0.069*	
H17B	0.4778	0.3459	0.9722	0.069*	
C18	0.6302 (3)	−0.2196 (3)	0.7780 (3)	0.0480 (9)	
C19	0.8761 (3)	0.4349 (3)	0.4953 (3)	0.0472 (9)	
H19	0.9325	0.4239	0.4717	0.057*	
C20	0.7118 (3)	0.3063 (3)	0.8838 (3)	0.0481 (9)	
H20A	0.7607	0.3161	0.8416	0.058*	
H20B	0.7317	0.2591	0.9340	0.058*	
C21	0.7095 (2)	0.4694 (3)	0.5638 (3)	0.0424 (8)	
C22	0.5545 (2)	0.2448 (3)	0.8952 (3)	0.0464 (9)	
H22A	0.5046	0.2128	0.8609	0.056*	
H22B	0.5710	0.1992	0.9478	0.056*	
C23	0.6903 (3)	−0.2364 (3)	0.7077 (3)	0.0520 (10)	
H23	0.7020	−0.3044	0.6866	0.062*	
C24	0.8283 (3)	0.5214 (3)	0.4682 (3)	0.0501 (10)	
C25	0.8969 (2)	0.2760 (3)	0.5893 (3)	0.0460 (9)	
H25	0.9546	0.2760	0.5675	0.055*	
C26	0.7325 (3)	−0.1511 (3)	0.6696 (3)	0.0497 (9)	
C29	0.6047 (3)	0.4040 (4)	0.9854 (3)	0.0651 (12)	
H29A	0.6191	0.3626	1.0414	0.078*	
H29B	0.5872	0.4740	1.0056	0.078*	
C31	0.9466 (3)	0.1246 (3)	0.6765 (4)	0.0587 (11)	
H31	0.9164	0.0617	0.6999	0.070*	
C32	0.6862 (3)	0.4125 (3)	0.9258 (3)	0.0644 (12)	
H32A	0.7356	0.4393	0.9643	0.077*	
H32B	0.6745	0.4626	0.8751	0.077*	
C33	0.9997 (4)	0.1702 (4)	0.7576 (4)	0.0823 (16)	
H33A	0.9601	0.1895	0.8075	0.099*	
H33B	1.0298	0.2337	0.7372	0.099*	
C34	1.1256 (3)	0.0495 (5)	0.7212 (4)	0.0892 (17)	
H34A	1.1637	−0.0058	0.7471	0.107*	
H34B	1.1634	0.1060	0.6994	0.107*	
C35	1.0070 (4)	0.0888 (6)	0.6008 (4)	0.107 (2)	
H35A	1.0392	0.1489	0.5767	0.129*	
H35B	0.9720	0.0583	0.5491	0.129*	
C36	1.0692 (4)	0.0900 (5)	0.7957 (4)	0.102 (2)	
H36A	1.1064	0.1237	0.8440	0.122*	
H36B	1.0387	0.0315	0.8247	0.122*	
C37	1.0730 (5)	0.0069 (6)	0.6402 (5)	0.124 (3)	
H37A	1.0407	−0.0552	0.6600	0.149*	
H37B	1.1128	−0.0143	0.5911	0.149*	
N1	0.66375 (19)	0.1590 (2)	0.7834 (2)	0.0378 (6)	
N2	0.87612 (19)	0.1999 (2)	0.6432 (2)	0.0453 (7)	
O1	0.71582 (17)	0.31273 (19)	0.65115 (19)	0.0460 (6)	
O2	0.76509 (17)	0.02931 (19)	0.66432 (19)	0.0481 (6)	
S1	0.57697 (10)	−0.32763 (9)	0.82770 (9)	0.0706 (4)	
H1	0.6038	−0.3393	0.9076	0.106*	
S2	0.80792 (11)	−0.17094 (9)	0.58054 (12)	0.0845 (5)	
H2	0.7681	−0.1853	0.5070	0.127*	
S3	0.87207 (8)	0.61165 (11)	0.38901 (9)	0.0714 (4)	
H3	0.8813	0.5694	0.3142	0.107*	
S4	0.60444 (7)	0.49280 (9)	0.60717 (10)	0.0643 (3)	
H4	0.5842	0.4215	0.6580	0.096*	
Zn1	0.75466 (3)	0.17547 (3)	0.68717 (3)	0.04322 (17)	

### Atomic displacement parameters (Å^2^)

**Table:**

	*U*^11^	*U*^22^	*U*^33^	*U*^12^	*U*^13^	*U*^23^
C8	0.0371 (19)	0.0402 (19)	0.0397 (18)	−0.0044 (15)	0.0040 (14)	−0.0043 (15)
C10	0.0411 (19)	0.0360 (18)	0.0413 (18)	0.0088 (15)	0.0077 (15)	−0.0006 (15)
C11	0.0378 (18)	0.0349 (18)	0.0438 (19)	−0.0001 (14)	−0.0010 (15)	0.0021 (15)
C12	0.0380 (18)	0.0345 (18)	0.047 (2)	−0.0001 (14)	0.0005 (15)	−0.0001 (15)
C13	0.0380 (19)	0.042 (2)	0.0429 (19)	0.0027 (15)	0.0079 (15)	0.0031 (15)
C14	0.050 (2)	0.042 (2)	0.048 (2)	−0.0052 (16)	−0.0065 (17)	0.0066 (17)
C15	0.043 (2)	0.0346 (18)	0.0392 (18)	−0.0041 (15)	0.0018 (15)	−0.0030 (15)
C16	0.044 (2)	0.045 (2)	0.049 (2)	−0.0040 (16)	0.0066 (16)	0.0029 (17)
C17	0.053 (2)	0.062 (3)	0.059 (3)	0.018 (2)	0.014 (2)	−0.006 (2)
C18	0.053 (2)	0.038 (2)	0.053 (2)	−0.0064 (17)	0.0002 (18)	0.0067 (17)
C19	0.043 (2)	0.052 (2)	0.046 (2)	−0.0131 (17)	0.0055 (16)	0.0000 (17)
C20	0.045 (2)	0.043 (2)	0.056 (2)	0.0041 (16)	0.0034 (17)	−0.0026 (18)
C21	0.044 (2)	0.0377 (19)	0.046 (2)	0.0011 (15)	0.0013 (16)	−0.0031 (16)
C22	0.040 (2)	0.050 (2)	0.050 (2)	0.0032 (16)	0.0093 (16)	−0.0022 (17)
C23	0.059 (2)	0.032 (2)	0.064 (3)	−0.0017 (17)	0.001 (2)	−0.0028 (18)
C24	0.054 (2)	0.052 (2)	0.043 (2)	−0.0160 (18)	−0.0063 (17)	0.0120 (18)
C25	0.0343 (19)	0.043 (2)	0.061 (2)	−0.0053 (16)	0.0097 (16)	−0.0027 (18)
C26	0.048 (2)	0.043 (2)	0.058 (2)	0.0019 (17)	0.0101 (18)	−0.0048 (18)
C29	0.071 (3)	0.063 (3)	0.061 (3)	0.012 (2)	0.008 (2)	−0.021 (2)
C31	0.034 (2)	0.041 (2)	0.101 (3)	0.0021 (16)	0.004 (2)	0.009 (2)
C32	0.073 (3)	0.051 (3)	0.070 (3)	0.001 (2)	0.006 (2)	−0.017 (2)
C33	0.106 (4)	0.085 (4)	0.055 (3)	0.034 (3)	−0.006 (3)	0.004 (3)
C34	0.057 (3)	0.110 (5)	0.100 (4)	0.027 (3)	−0.007 (3)	0.025 (4)
C35	0.106 (4)	0.138 (5)	0.077 (3)	0.078 (4)	−0.021 (3)	−0.032 (3)
C36	0.123 (5)	0.110 (5)	0.070 (4)	0.041 (4)	−0.025 (3)	0.015 (3)
C37	0.107 (5)	0.155 (7)	0.108 (5)	0.093 (5)	−0.032 (4)	−0.044 (5)
N1	0.0361 (15)	0.0359 (16)	0.0417 (16)	0.0047 (12)	0.0059 (12)	−0.0004 (13)
N2	0.0348 (16)	0.0352 (16)	0.066 (2)	0.0015 (12)	0.0069 (14)	−0.0013 (15)
O1	0.0450 (14)	0.0365 (13)	0.0574 (16)	0.0053 (11)	0.0188 (12)	0.0088 (12)
O2	0.0472 (14)	0.0342 (13)	0.0639 (16)	−0.0003 (11)	0.0191 (12)	−0.0018 (12)
S1	0.0982 (10)	0.0420 (6)	0.0725 (8)	−0.0164 (6)	0.0176 (7)	0.0121 (5)
S2	0.1057 (11)	0.0464 (7)	0.1048 (11)	0.0007 (6)	0.0616 (9)	−0.0149 (6)
S3	0.0696 (8)	0.0765 (8)	0.0678 (7)	−0.0230 (6)	−0.0010 (6)	0.0353 (6)
S4	0.0503 (6)	0.0478 (6)	0.0960 (9)	0.0140 (5)	0.0214 (6)	0.0098 (6)
Zn1	0.0413 (3)	0.0359 (3)	0.0531 (3)	0.00146 (17)	0.01108 (19)	0.00153 (18)

### Geometric parameters (Å, °)

**Table:**

C8—C19	1.408 (5)	C23—H23	0.9300
C8—C15	1.426 (5)	C24—S3	1.745 (4)
C8—C25	1.434 (5)	C25—N2	1.275 (5)
C10—N1	1.488 (4)	C25—H25	0.9300
C10—C20	1.520 (5)	C26—S2	1.741 (4)
C10—C22	1.532 (5)	C29—C32	1.513 (6)
C10—H10	0.9800	C29—H29A	0.9700
C11—C16	1.400 (5)	C29—H29B	0.9700
C11—C12	1.419 (5)	C31—N2	1.491 (5)
C11—C13	1.441 (5)	C31—C33	1.496 (7)
C12—O2	1.295 (4)	C31—C35	1.497 (7)
C12—C26	1.410 (5)	C31—H31	0.9800
C13—N1	1.278 (4)	C32—H32A	0.9700
C13—H13	0.9300	C32—H32B	0.9700
C14—C21	1.370 (5)	C33—C36	1.542 (7)
C14—C24	1.379 (6)	C33—H33A	0.9700
C14—H14	0.9300	C33—H33B	0.9700
C15—O1	1.286 (4)	C34—C36	1.467 (8)
C15—C21	1.415 (5)	C34—C37	1.476 (9)
C16—C18	1.361 (5)	C34—H34A	0.9700
C16—H16	0.9300	C34—H34B	0.9700
C17—C29	1.509 (6)	C35—C37	1.530 (7)
C17—C22	1.519 (5)	C35—H35A	0.9700
C17—H17A	0.9700	C35—H35B	0.9700
C17—H17B	0.9700	C36—H36A	0.9700
C18—C23	1.382 (6)	C36—H36B	0.9700
C18—S1	1.745 (4)	C37—H37A	0.9700
C19—C24	1.359 (6)	C37—H37B	0.9700
C19—H19	0.9300	Zn1—N1	1.971 (3)
C20—C32	1.525 (5)	Zn1—N2	1.971 (3)
C20—H20A	0.9700	Zn1—O1	1.899 (2)
C20—H20B	0.9700	Zn1—O2	1.886 (2)
C21—S4	1.737 (4)	S1—H1	1.2000
C22—H22A	0.9700	S2—H2	1.2000
C22—H22B	0.9700	S3—H3	1.2000
C23—C26	1.371 (6)	S4—H4	1.2000
			
C19—C8—C15	120.5 (3)	C17—C29—C32	111.4 (4)
C19—C8—C25	117.5 (3)	C17—C29—H29A	109.4
C15—C8—C25	121.9 (3)	C32—C29—H29A	109.4
N1—C10—C20	108.7 (3)	C17—C29—H29B	109.4
N1—C10—C22	115.9 (3)	C32—C29—H29B	109.4
C20—C10—C22	110.0 (3)	H29A—C29—H29B	108.0
N1—C10—H10	107.3	N2—C31—C33	110.7 (4)
C20—C10—H10	107.3	N2—C31—C35	114.0 (4)
C22—C10—H10	107.3	C33—C31—C35	110.3 (4)
C16—C11—C12	120.3 (3)	N2—C31—H31	107.2
C16—C11—C13	117.0 (3)	C33—C31—H31	107.2
C12—C11—C13	122.7 (3)	C35—C31—H31	107.2
O2—C12—C26	119.6 (3)	C29—C32—C20	111.9 (4)
O2—C12—C11	124.5 (3)	C29—C32—H32A	109.2
C26—C12—C11	115.9 (3)	C20—C32—H32A	109.2
N1—C13—C11	127.1 (3)	C29—C32—H32B	109.2
N1—C13—H13	116.4	C20—C32—H32B	109.2
C11—C13—H13	116.4	H32A—C32—H32B	107.9
C21—C14—C24	118.9 (4)	C31—C33—C36	110.8 (4)
C21—C14—H14	120.5	C31—C33—H33A	109.5
C24—C14—H14	120.5	C36—C33—H33A	109.5
O1—C15—C21	119.9 (3)	C31—C33—H33B	109.5
O1—C15—C8	124.7 (3)	C36—C33—H33B	109.5
C21—C15—C8	115.4 (3)	H33A—C33—H33B	108.1
C18—C16—C11	120.7 (3)	C36—C34—C37	112.4 (5)
C18—C16—H16	119.7	C36—C34—H34A	109.1
C11—C16—H16	119.7	C37—C34—H34A	109.1
C29—C17—C22	110.8 (3)	C36—C34—H34B	109.1
C29—C17—H17A	109.5	C37—C34—H34B	109.1
C22—C17—H17A	109.5	H34A—C34—H34B	107.9
C29—C17—H17B	109.5	C31—C35—C37	110.1 (5)
C22—C17—H17B	109.5	C31—C35—H35A	109.6
H17A—C17—H17B	108.1	C37—C35—H35A	109.6
C16—C18—C23	120.9 (4)	C31—C35—H35B	109.6
C16—C18—S1	119.9 (3)	C37—C35—H35B	109.6
C23—C18—S1	119.2 (3)	H35A—C35—H35B	108.2
C24—C19—C8	120.3 (4)	C34—C36—C33	112.2 (4)
C24—C19—H19	119.9	C34—C36—H36A	109.2
C8—C19—H19	119.9	C33—C36—H36A	109.2
C10—C20—C32	110.8 (3)	C34—C36—H36B	109.2
C10—C20—H20A	109.5	C33—C36—H36B	109.2
C32—C20—H20A	109.5	H36A—C36—H36B	107.9
C10—C20—H20B	109.5	C34—C37—C35	111.2 (5)
C32—C20—H20B	109.5	C34—C37—H37A	109.4
H20A—C20—H20B	108.1	C35—C37—H37A	109.4
C14—C21—C15	123.5 (3)	C34—C37—H37B	109.4
C14—C21—S4	119.2 (3)	C35—C37—H37B	109.4
C15—C21—S4	117.3 (3)	H37A—C37—H37B	108.0
C17—C22—C10	109.0 (3)	C13—N1—C10	120.0 (3)
C17—C22—H22A	109.9	C13—N1—Zn1	123.3 (2)
C10—C22—H22A	109.9	C10—N1—Zn1	116.7 (2)
C17—C22—H22B	109.9	C25—N2—C31	119.3 (3)
C10—C22—H22B	109.9	C25—N2—Zn1	123.6 (3)
H22A—C22—H22B	108.3	C31—N2—Zn1	117.1 (2)
C26—C23—C18	118.9 (4)	C15—O1—Zn1	127.1 (2)
C26—C23—H23	120.5	C12—O2—Zn1	127.7 (2)
C18—C23—H23	120.5	C18—S1—H1	109.5
C19—C24—C14	121.3 (4)	C26—S2—H2	109.5
C19—C24—S3	120.2 (3)	C24—S3—H3	109.5
C14—C24—S3	118.5 (3)	C21—S4—H4	109.5
N2—C25—C8	127.0 (3)	O2—Zn1—O1	151.46 (13)
N2—C25—H25	116.5	O2—Zn1—N1	94.46 (11)
C8—C25—H25	116.5	O1—Zn1—N1	93.98 (11)
C23—C26—C12	123.2 (4)	O2—Zn1—N2	90.96 (12)
C23—C26—S2	119.4 (3)	O1—Zn1—N2	92.96 (12)
C12—C26—S2	117.4 (3)	N1—Zn1—N2	154.63 (14)
